# End-of-Life Decision-Making in Pediatric and Neonatal Intensive Care Units in Croatia—A Focus Group Study among Nurses and Physicians

**DOI:** 10.3390/medicina58020250

**Published:** 2022-02-07

**Authors:** Filip Rubic, Marko Curkovic, Lovorka Brajkovic, Bojana Nevajdic, Milivoj Novak, Boris Filipovic-Grcic, Julije Mestrovic, Kristina Lah Tomulic, Branimir Peter, Ana Borovecki

**Affiliations:** 1 Department for Pediatrics, University Hospital Centre, 10000 Zagreb, Croatia; filiprubic@gmail.com (F.R.); mnovak@kbc-zagreb.hr (M.N.); borisfilipovicgrcic@gmail.com (B.F.-G.); 2 University Psychiatric Hospital Vrapče, 10000 Zagreb, Croatia; markocurak@gmail.com; 3 School of Medicine, University of Zagreb, 10000 Zagreb, Croatia; bojana.nevajdic@hotmail.com; 4 Department for Psychology, Faculty of Croatian Studies, University of Zagreb, 10000 Zagreb, Croatia; lovorka.brajkovic@yahoo.com; 5 Department for Paediatrics, University Hospital Centre Split, 21000 Split, Croatia; julije.mestrovic@gmail.com; 6 Department for Pediatrics, University Hospital Centre Rijeka, 51000 Rijeka, Croatia; klahtomulic@gmail.com; 7 Department for Gynecology and Obstetrics, University Hospital Centre Rijeka, 51000 Rijeka, Croatia; branimir.peter@gmail.com; 8 Andrija Stampar School of Public Health, School of Medicine, University of Zagreb, Rockefellerova 4, 10000 Zagreb, Croatia

**Keywords:** pediatric intensive care unit, neonatal intensive care unit, critical care, end-of-life, nurses, physicians, focus groups

## Abstract

*Background and Objectives:* Working in pediatric and neonatal intensive care units (ICUs) can be challenging and differs from work in adult ICUs. This study investigated for the first time the perceptions, experiences and challenges that healthcare professionals face when dealing with end-of-life decisions in neonatal intensive care units (NICUs) and pediatric intensive care units (PICUs) in Croatia. *Materials and Methods:* This qualitative study with focus groups was conducted among physicians and nurses working in NICUs and PICUs in five healthcare institutions (three pediatric intensive care units (PICUs) and five neonatal intensive care units (NICUs)) at the tertiary level of healthcare in the Republic of Croatia, in Zagreb, Rijeka and Split. A total of 20 physicians and 21 nurses participated in eight focus groups. The questions concerned everyday practices in end-of-life decision-making and their connection with interpersonal relationships between physicians, nurses, patients and their families. The constant comparative analysis method was used in the analysis of the data. *Results:* The analysis revealed two main themes that were the same among the professional groups as well as in both NICU and PICU units. The theme “critical illness” consisted of the following subthemes: the child, the family, myself and other professionals. The theme “end-of-life procedures” consisted of the following subthemes: breaking point, decision-making, end-of-life procedures, “spill-over” and the four walls of the ICU. The perceptions and experiences of end-of-life issues among nurses and physicians working in NICUs and PICUs share multiple common characteristics. The high variability in end-of-life procedures applied and various difficulties experienced during shared decision-making processes were observed. *Conclusions:* There is a need for further research in order to develop clinical and professional guidelines that will inform end-of-life decision-making, including the specific perspectives of everyone involved, and the need to influence policymakers.

## 1. Introduction

Caring for a critically ill child in the context of an intensive care unit (ICU) is a cognitively and emotionally challenging task [1,2,3]. The needs of highly stressed parents, the needs of the critically ill child and the omnipresence of death and dying interrelate and place a complex matrix of personal and professional demands on healthcare providers [2,4,5,6,7]. Additionally, critically ill patients often require fast-paced, complex and precise care [8,9,10,11]. The most challenging of all issues that occur within neonatal intensive care units (NICUs) and pediatric intensive care units (PICUs) are those related to end of life [1,2,4,6,12,13]. Even though end-of-life decisions commonly occur (almost on a daily basis), healthcare professionals often feel inadequately prepared for them [2,7,14,15]. The intricacy of end-of-life decisions adds additional layers of complexity, and creates additional demands in already demanding work. Additionally, the broader sociocultural context exerts a significant and unavoidable influence on end-of-life issues, informing and influencing the underlying values and beliefs of everyone involved [13,16,17,18]. The literature signals that this could be a critical factor in the high and growing incidence of burnout among healthcare professionals in NICUs and PICUs, which has a significant and detrimental influence on the quality and safety of care, as well as on different patient, family member and health professional outcomes [2,4,19]. Therefore, there is a great need to research issues related to end-of-life care in NICUs and PICUs, considering the specific influences of the sociocultural context.

The Croatian situation regarding end-of-life decision-making in pediatric and neonatal ICUs is not without its challenges. According to the data collected by one of the authors of this paper, there are seven NICUs with 76 intensive care beds and 42 post-intensive care beds with 48 physicians and 204 nurses on staff in Croatia. There are five PICUs with 49 intensive care beds and five post-intensive care beds with 24 physicians and 121 nurses on staff. All of these PICUs and NICUs can be found in the healthcare institutions at the tertiary level of healthcare. The survey of Croatian ICUs by Degoricija et al. observed a shortage of trained medical and nursing staff in ICUs [20]. The permanent staff of the PICUs and NICUs are physicians and nurses. If there is a need for social workers, psychiatrists or psychologists or clergymen, they can be called for. However, they are not part of the PICU and NICU staff. Social workers in Croatian hospitals are usually attached to a hospital or to departments, e.g., pediatric, psychiatric departments. Psychiatrists and psychologists are usually attached to psychiatric departments. There is usually a hospital Catholic chaplain attached to larger hospitals or sometimes clergymen are called from the religious congregations of the patients. There are no resources available to support the physicians and nurses, such as counseling, referrals to mental health services, addressing burnout, etc.

When it comes to physician–child–parent interactions within the provision of healthcare in Croatia, they are mainly regulated by the Family Act (NN 103/15, 98/19). According to Article 88 of the Family Act, informed consent is given by a parent or legal guardian of the child. The legal age for informed consent is 18. However, children of the age of 16 can give informed consent for medical procedures, but only for those medical treatments that do not entail risks of severe consequences for the physical or mental health of the child. Only in an emergency situation, when the parent or legal guardian is not present, can a medical procedure be performed on a child without informed consent. If there is a discrepancy between the parents’, the physicians’ and the child’s opinions about a medical procedure, there is the possibility of the initiation of a special non-litigious court procedure aimed at protecting the child’s welfare (the Family Act Article 135). Any suspicion of child abuse has to be reported immediately by healthcare workers (the Law on Protection against Domestic Violence, Article 7 (NN 70/17, 126/19, 84/21)). Children who are younger than 16 years of age have a right to assent (express their agreement or disagreement with a medical procedure) and this will be taken into account [21].

There are no professional guidelines on end-of-life decision-making in any ICU in Croatia. The following statement can only be found in the Code of Medical Ethics and Deontology of the Croatian Medical Chamber and the Croatian Medical Association: “Continuing intensive treatment of a patient in an irreversible final condition is not medically necessarily well-founded, and excludes the right of the dying patient to a dignified death.”

No forms of anticipatory decision-making (e.g., living wills, do-not-resuscitate orders) by patients are regulated by law in Croatia, except in the Act on Human Organ Transplantation for Medical Purposes (NN 144/12), where anticipatory decision-making is permitted if a person wishes to opt out from the donor pool. The death of a person can be declared on the basis of neurologic criteria (brain death) [22,23].

Research by Sorta-Bilajac et al. indicated that the most frequent ethical dilemmas that Croatian physicians and nurses face in everyday practice are connected to the uncertain or impaired decision-making capacity of patients, and withdrawing or withholding treatment at the end of life [24]. There has never been any in-depth research regarding end-of-life decision-making in pediatric and neonatal ICUs in Croatia.

In 2017, a project funded by the Croatian Science Foundation was launched, entitled “Values and Decisions at the End of Life” (VAL-DE-END), which aimed to investigate end-of-life decision-making processes in ICUs in tertiary-level healthcare in the Republic of Croatia, through a series of retrospective and prospective studies, of the quantitative and qualitative types. These tertiary-healthcare-level intuitions were chosen because of the complexity of the cases that they deal with in their everyday practice. The project is led by a multidisciplinary research team, comprising philosophers, theologians, ICU physicians (including those working in pediatric and neonatal ICUs), lawyers, psychologists, psychiatrists and palliative care experts. It has two tracks, one dealing with adult ICUs and one dealing with pediatric ICUs, as these are two very different settings, with different sets of issues at stake. The proposed project methodology had already been tested in a similar study by Grosek et al. in the neighboring country of Slovenia, with which Croatia shares a common sociocultural background and past [25]. One of the outputs of the project is a proposal of guidelines for end-of-life decision-making in intensive care units in Croatia (pediatric, neonatal and adult), similar to those already proposed in Slovenia [26]. Two project team members were invited to participate in the work of a recently formed working group on a national level that will be involved in drafting end-of-life guidelines at the national level.

This focus group study is part of this project. It is also the first study of pediatric and neonatal intensive care units in Croatia focusing on end-of-life issues. The main aim of this focus group study was to study decision-making related to end of life in pediatric and neonatal ICUs in tertiary-level healthcare institutions in the Republic of Croatia, from the perspective of front-line healthcare professionals. More specifically, our focus was on the everyday experience of physicians and nurses working in NICUS and PICUs regarding end-of-life decision-making: the types of decisions made, the involvement of the family and patient (if applicable) in decision-making, communication between team members and family and team members, the dynamics of the process of the end-of-life decision-making and the burdens that physicians and nurses are faced with as a result of the outcomes of this process and their coping strategies. We were also interested in the challenges that physicians and nurses face when it comes to technical and organizational issues, as well as the challenges that they face in their interprofessional and intraprofessional relationships in the process and as the result of the process of end-of-life decision-making. Our hypothesis was that end of-life decision-making in Croatian NICUs and PICUs presents physician and nurses with difficult challenges and burdens due to the complex organizational and interpersonal issues raised.

## 2. Materials and Methods

### 2.1. Setting

The study was undertaken at three distinct research sites, Zagreb, Rijeka and Split in the Republic of Croatia, and involved healthcare staff participants from a total of five institutions (altogether, five NICUs and three PICUs) at the tertiary level of healthcare. This study was conducted according to the guidelines of the Declaration of Helsinki and approved by all of the ethics committees. We also wanted to include participants from the fourth largest city in Croatia, Osijek, which has also a tertiary healthcare institution. However, the approval of the ethics committee of Osijek Clinical Medical Center was never received, nor did we receive any reply to our officially submitted request with the supporting documents. The focus group discussions were conducted between December 2018 and July 2019.

### 2.2. Participants

Intensive care physicians, with a minimum of one year’s working experience as specialists, and registered nurses, also with a minimum of one year’s working experience, currently working in NICUs or PICUs, were invited to participate, with the aim of obtaining detailed interdisciplinary opinions. The trainees were excluded because we wanted to have medical professionals with sufficient experience and knowledge about the end-of-life procedures and decision-making. We also tried to include an equal number of participants in each focus group, where this was possible, according to gender, seniority (more or less than five years’ working experience in NICUs and PICUs) and type of ICU unit (NICU or PICU). The members of the project team, who were physicians in pediatric and neonatal ICUs, approached their colleagues and asked them to ask nurses and physicians who were eligible for the focus group research, according to our inclusion criteria, to participate in the research. Participation in the research was voluntary and anonymous. The sample frame focused on generating a sample size that would produce high-quality data, which was achieved by purposive sampling [27]. Purposive sampling allowed the researchers to select participants who were able to provide perspectives that specifically related to the topic of inquiry [28]. Focus groups were conducted separately with physicians and nurses. The focus group participants were physicians and nurses, since they were staff in the pediatric and neonatal ICUs in Croatia, while other healthcare professionals, e.g., physiotherapists, are not part of the ICU departments but attend on a regular basis from other departments [20]. A total of 20 physicians and 21 nurses responded positively to the invitation to participate in the focus group sessions. All of them were educated in Croatia. Only 3 of them reported having additional education (usually for several months or a year in an ICU department) outside of Croatia (USA and EU). The structure of focus group participants can be seen in Table 1.

### 2.3. Procedure

The focus groups were conducted by three focus group moderators (F.R., M.C. and L.B.). A semi-structured discussion guide was used, with stimulating questions, developed after a systematic review of the current literature, discussions within the research project team and extensive pilot testing (Table 2). During the focus group sessions, the interviewers probed participants for their interpretation of the questions. They were probed on how they formulated their responses to the questions.

A total of eight focus group discussions were conducted, one per professional group at every research site, except in Rijeka, where a total of four focus groups were held (two for both professional groups for logistic reasons, since the pediatric and neonatal ICUs were not at the same location). All study participants gave written informed consent, after being fully informed about the specific and overall research project methodology and goals. Participant confidentiality was upheld as no identifiable information was collected. All focus group discussions were conducted in the local language. Data were audio-recorded and transcribed verbatim. Audio recordings and transcripts were reviewed repeatedly to ensure that precise information was transcribed. All collected data were anonymized and stored according to the Croatian Law on Data Protection (NN 106/2012) and EU Data Protection Directive 95/46/EC. Ethical approval was obtained from the ethics committees of all the institutions involved in the research.

### 2.4. Data Analysis

Data were analyzed using the constant comparative analysis method, informed by constructivist grounded theory and grounded theory methods [27,28,29,30]. The transcripts of the focus group discussions were coded by reading each document and attributing codes to sentences, paragraphs or sections. The coding was done by F.R., M.C. and L.B., who conducted the focus group discussions. Inter-coder agreement was assessed between three coders to compare the consistency of code use and rectify discrepancies before the whole data set was coded. Data analysis was inductive, in order to understand individual views and perceptions. New codes were added during subsequent reading of transcripts and data, when it was not initially clear how they should be coded. The coded sections established were compared with similarly coded segments to ensure consistency. After the final coding was completed, files were compiled with the title of the code, and stored in files labelled with each code. The codes that had common elements were merged to form subthemes and main themes. The reconstruction of the data was then presented to the project team members, who were physicians and experts in pediatric and neonatal intensive care medicine, and who were also present during the focus group discussions, in order to determine whether the proposed data reconstruction was a reasonable account of the discussion that took place in the focus groups [31].

## 3. Results

The analysis revealed two main themes that were the same in both professional groups, as well as in both NICU and PICU units: critical illness and end of life. Some differences between professional groups and units did occur, and these are discussed below. An overview of the main themes, subthemes and related codes that were identified from the analysis of the focus groups is presented in Table 3.

### 3.1. The Critical Illness

The major theme “the critical illness” consisted of the following subthemes: the child, the family, myself and other professionals, and related codes.

#### 3.1.1. The Child

This subtheme emerged from both physicians’ and nurses’ discussions about the clinical and prognostic uncertainties that are omnipresent in the context of NICUs and PICUs. All participants in the focus group discussions touched upon this subtheme in one way or another. They highlighted the need for the constant monitoring and reevaluation of clinical decisions made previously, as is shown in the following statement by a physician:

*We always say this child should be treated to some extent. As long as we see that there is improvement in some sense and there is any hope that it could be better we give it our all. The moment we see that everything is spinning in a circle and that the child is generally getting worse and worse, then I think we need to draw some sort of limit and start talking to the parents about the child not surviving whatever we do*.

Interestingly, this uncertainty was mentioned to a similar extent in both groups of professionals, as well as by participants from distinct units and institutions.

One nurse told the following story:

*I had a case where 3 pediatricians, and 3 neonatology nurses really worked on a child for 20 min and saw the child slipping away…, they tried everything: central venous catheter, resuscitation, intraosseous administration of fluids, everything, and we didn’t give up. It lasted for 1.5 h. Mom watched everything and saw that we saved that child and he is home today. And I thought… it was a miracle.*

One physician came up with a similar case:

*I am now near the end of my career. I had one patient, a small patient who was practically dead, but I fought for him. …the recommendations say you have to perform …minutes of resuscitation and after that you stop. However, I continued the reanimation and after …minutes of resuscitation I gave 3 doses of adrenaline and then I told the nurses that it was enough and that we should stop now. I looked at the patient and he was breathing; He had come back to life… So a totally wrong estimation, after 38 years of experience, I transferred him later ………and he survived, he was… without brain injury. Totally wrong…, I declared the child had no chances of survival and he survived…*

The primacy of the highly vulnerable patients’ best interests was unquestioned, although, when considered in the context of overwhelming uncertainties and the awareness of the inappropriateness and futility of certain interventions, it could not always be clearly identified.

This is nicely summed up in a statement given by one physician:

*The first decision that we have to make when it comes to a child is primarily the quality of life of that child before the onset of deterioration of their condition. We have to take into account whether the child has an incurable chronic disease, whether the child has suffered or had any pain during their life and how he or she lived their life until intensive care. The decisions we will make are the following: whether we will resuscitate or not; whether we will initiate any form of therapy, or will we just continue with the basic support, which in my opinion every child should have: pain relief, hydration and caloric intake; whether we will continue with some earlier therapy and then only perform resuscitation.*

The difference that was most prominent between neonatal and pediatric ICUs was the patient’s age, as the level of dependence was singled out as the most important indicator of vulnerability. This was evident from a statement by one neonatologist: *…No active resuscitation is performed; the child is not intubated, only placed in a thermoneutral environment. This only applies for children under the 22nd week of gestational age…*

#### 3.1.2. The Family

This subtheme is another that was present in the discussions of all focus group participants in one way or another. Within all group discussions, it was firmly recognized that different individuals and groups have different ideas on the use of medical technologies. Thus, parents and healthcare providers may have different notions regarding the provision of life-sustaining interventions. Parents base their decisions on many factors, mostly not related to probabilities. Additionally, the values used for decision-making vary widely between patients’ families and multiple team members, leading to tension about “right” and “wrong”. The influence of the different and variable psychosocial needs of the parents was recognized, and they may often create a certain amount of tension between the perceived best interests of the patient and the parents’ wishes. Specific Croatian circumstances were also discussed, specifically related to the more paternalistic approach to the involvement of the family in decision-making:

*I explain the situation as it is to the parents. I think that parents in Croatia, and we as a nation, are not sufficiently informed about or aware of the facts of life and death. Neither are parents able to accept loss of a child, nor to accept any, conditionally speaking, responsibility. Because when you ask them what they think (what should we do), no one will say stop reviving my child, i.e., very few will be brave enough to understand that. When I talk to parents I tell them that what I can promise them is that their child will not suffer at any point. I will do absolutely everything. We are all here and we will all do absolutely everything to keep their child from suffering. And then if I see that it’s coming to an end, then somehow I say I think any extra intervention would cause your child extra pain without actually getting what we all want together, and that’s your living and healthy child.*

The role of emotions, regret, hope, quality of life, resilience and relationships was often discussed, as can be seen from the following statement by a neonatologist:

*Even if one just sits quietly with them and listens to their stories about that child and what he was like and what happened… sometimes it is enough…. they tell you everything that troubles them, especially, if an accident has occurred and the parents feel guilty about it. That is terribly important. It is important that someone is next to them, that he will help them with that… to realize that they are not really to blame. A lot happens and parents blame themselves, and it’s very hard to live like that. That’s why support is so important and we’ve seen from these examples of ours that it really helps.*

However, the parents’ presence and involvement were seen as crucial, as well as beneficial, although their presence can also often contribute to tension (e.g., caring for the psychological functioning of patients versus the psychosocial needs of the parents). The emphasis was also on the very act of handing over the care of the child by the parents to the healthcare staff, which is full of meaning and responsibility. The unanimous conclusion was that end-of-life discussions with parents should be individualized and personalized, as can be seen from a statement by a pediatrician:

*I think our assessment and attitude is also very important, compared to other departments, we take a lot of time to talk to parents and we have a lot of mutual trust. We are quite open about what we do, what our expectations are about the patient, what we think if the child is extremely poorly, and then we tell them that it is so, and when there is hope for him as well. And they see that we are quite homogeneous as doctors. This good and open relationship with parents makes it much easier for us, and parents feel that there is a limit. And indeed if the child is seriously ill we do not give up, but give the maximum, they come from home and they see it. Then they respect our decision, but then we ask them to say what they think about it now. But there is also positive feedback from the other side and that is why I think there is no problem. And in the end we hug each other, we cry, people are grateful for everything we have given them. And they find relief in the decision when they say they think it makes no sense to resuscitate anymore, because the child will no longer be the same person they were before the illness.*

The spiritual needs of families are also important and are often met by healthcare teams, as can been seen from the statement by one nurse:

*No matter what religion our parents are, we allow all the holy sacraments to be administered and priests and others are allowed to come. I say that I am a Roman Catholic and that I pray for them with all my heart, whether they are Orthodox, whether they come from Bosnia, Serbia, Albania. Visits by imams are allowed as well as all different religious rites and baptisms if they are asked for. We call it a Capuchin monastery and we have been godparents 20–50 times…*

#### 3.1.3. Myself

The challenge for healthcare professionals (and this was more present among the participating physicians 12 physicians vs. 10 nurses) is to remain compassionate and emotionally available to the parents and families while, at the same time, keeping enough emotional distance to remain objective and “technically” competent. In other words, it is not easy to find a place on the continuum between empathy and detachment. This, among other things (for example, the inability to find closure, and the grief after the death of a patient), creates distress, and involves significant emotional effort, which can result in exhaustion, fatigue, moral distress and eventually burnout, as can be seen from the statement by a neonatologist:

*We mostly state here how hard it is for us, it is really hard for us, I would say that as life goes on, it is getting harder and harder to make such decisions, and on the other hand when decisions have already been made based on some team opinion… one may not want to participate in it because it is emotionally difficult for him or her …*

*…Burnout wears us down and then we wonder: why does my head hurt, when I am resting nicely, and looking at the sea and the fish is grilling on the grill and I start to remember things …We actually have PTSD… Every time I see a child with cerebral palsy in the town I think, God is it one of ours?…*

*…Man, it cuts me every time, and you don’t talk about it, when I go to the cemetery and look at the graves of little children…*

#### 3.1.4. Other Professionals

Within this subtheme, the greatest differences were perceived between the healthcare professional groups (8 nurses vs. 4 physicians), as well as the units. Nurses placed a higher emphasis on interprofessional relationships.

*…We have excellent cooperation with all the doctors on duty in the intensive care unit and they are with us, which is very important to us.*

Physicians placed more emphasis on intraprofessional relationships (more prominent in PICUs, as there is a greater need to consult with and consider the opinions of other specialists when making decisions), as can be seen from the statements by two physicians:

*…the three of us make a joint decision and stick to it. It’s good that there are three of us. The majority always wins. Everyone has their own arguments that are always taken into account, the only question is that apart from the three of us, there are cardiologists and endocrinologists on duty…*

*…I feel relief when we discuss the case and reach a common position. It can be difficult in during that discussion, but when you know that we have reached a consensus, which comes from us both professionally and humanly, it is as if that decision no longer bothers me afterwards…*

These relationships were mostly perceived as satisfactory, even though sometimes tensions did occur.

*When they say “I tried to reanimate him for a while”, it means to us that they tried to calm themselves and the parents… And that’s understandable to me because the child might have been in a better state at the time. What we do is negotiate everything, but we can never be 100% sure that others will respect it. In the end, it should be understood that this man is alone on duty with a dying child and a parent who is demanding something from him, and he may not be sticking to what we agreed at that point.*

The need was often stressed for shared goals and aims, understanding and perceptions among healthcare professionals involved in the care of an individual patient, as expressed by a physician:

*So there are a lot of these problems and we actually talk about the feelings that we have problems feeling… our young specialists in training are a particularly vulnerable group…*

However, the differing values and viewpoints between the professional groups, as well as between members of different medical specialties, were recognized and stressed as having a significant impact on the organization and provision of clinical care. Here, also, the importance of adequate leadership was often stressed, most commonly referring to the immediate medical (team) leaders, as expressed in these two statements by a physician who was also the head of the department and the team:

*…The head of the department has the final word, you have to be strong and then in a way it takes part of the stress off the others…*

### 3.2. End-of-Life

The major theme “the end-of-life process” consisted of the following subthemes: breaking point (addressed by 18 physicians and 9 nurses), decision-making (addressed by 18 physicians and 15 nurses), end-of-life procedures (addressed by 18 physicians and 12 nurses), “spill-over” (addressed by 11 physicians and 9 nurses) and the four walls of the ICU (addressed by 10 physicians and 11 nurses), and related codes.

#### 3.2.1. Breaking Point

Different barriers and enablers were discussed, relating to various personal, relational and organizational factors in the initiation of end-of-life discussions. The transition to end-of-life care was considered as not always straightforward. It seems that the most common “official initiator” of this discussion was the treating physician, as expressed by a nurse:

*The physicians themselves make the decision with each other first, and they present it to the parents, and we know the final agreement because they tell us what it is, they also tell the physicians on duty, so they tell us….*

*The extent to which certain treating physicians consider inputs from other health care team members (including other physicians and nurses), commonly referred to as “informal initiators”, was highly variable. However, nurses’ input in the decision-making process was considered to be highly valuable, and was facilitated by the physician members of the health care team: … Primarily it’s up to the physician, he’s the one who says it’s enough now, of course the nurse is present throughout that whole period, but after that the role of the nurse is very important…*

*…The team leader has to listen; practically I rely heavily on what the nurses say, how they think, and then you listen a little bit to other team members and what they say or think, communicate verbally or nonverbally, and then basically make a decision…*

#### 3.2.2. Decision-Making

The omnipresent interrelatedness of cognitive and emotional factors in the decision-making process, as well as recognizing the role of the underlying values of all involved, was one of the most commonly mentioned issues overall. Communicating medical uncertainties to the patients’ parents is especially demanding. Adequate involvement in end-of-life decisions requires the parents to comprehend complicated medical scenarios, as well as incorporation of the family’s values and goals, through a shared decision-making process, as stated by one physician:

*When we think that’s the case then we talk to the parents, of course we don’t say that we’re going to shut down the machines now, because we think it doesn’t make sense anymore, but we try to see what they think about their child’s fate… In other places, 1000 km to the west, it is done in a different way. It is much easier in Scandinavia than in our geographical position. However, the fact is that when we get the consent of the parents, and we see that the child will not survive, we do not even have to ask them to sign, …then we do not perform resuscitation when the time of death comes… We will not start resuscitation and that child will die, and we will give some sedative or analgesic to the child so they don’t suffer… and this we can always communicate and say that we will continue to treat pain and suffering… but that the child will die, and this should be said exactly in these words, that the child will die, so that it is clear to them.*

However, the definition of futile care and the process of determining the death of the patient (the fear of futility being declared prematurely and the fear of death being declared incorrectly was considered as inherent to human nature) can be seen from one physician’s experience:

*For me, one stressful situation is when you are treating a child intensively and you have made the decision that you will have to turn off the button. You are playing God, and that is really very difficult for me. On the other hand, again you are aware that the continuation of treatment that is not promising does not make sense, but you do not know whether something is promising or not. So the anguish about that patient is very difficult for me, but I have made a lot of decisions like that. When I have said that the child has died and we are done, we will not go any further, fortunately no one survived. I mean no one came to life, but it could have happened.*

Such situations create tension among healthcare professionals, and consequently also with family members. This kind of situation was considered to be rare, but highly distressing, commonly creating “intractable disagreements”. Most intractable disagreements occurred around “inappropriate” or “potentially inappropriate” treatments, which have at least some chance of accomplishing the effect sought by the patient/parents, but clinicians believe that competing ethical considerations justify not providing them. In this sense, withholding certain information was considered necessary within the process of shared decision-making about treatment options that are considered to be futile:

*I have been in a situation many times when the parents were not ready to say goodbye at that moment, I knew the child was dead but I kept the child on a ventilator and the parent thought the child was alive, and I waited for the moment when the parent was ready to say goodbye to the child. So I lied to the parent, God forgive me. I don’t think I’m a religious man, but I did do a good deed… did I prolong the suffering of that child to please the parent? I struggle with that, but I still think it was more important to please the parent than to prolong the child’s suffering.*

Several units reported having specific local guidelines on end-of-life decision-making, which they used only partially. Instead, adopting certain unwritten rules that are a part of the culture of a specific ICU, while still being able to uphold a certain degree of discretion in decision-making, was the prominent mode of behavior and practice in ICUs.

#### 3.2.3. End-of-Life Procedures

There were significant differences and high variability among the end-of-life procedures implemented in different institutions and units. Most participants used the term “comfort care”, as delineating situations where life-sustaining treatment would normally/ideally be withdrawn. The term “symbolic care” was used to describe the provision of certain interventions as an act of sincere caring and compassion, mostly concerning the needs of the parents, that have questionable benefit for the patient themselves (different benchmarks were used here on which procedures could be considered symbolic). Often, these were perceived as necessary in order to provide closure to the family members.

*…Maybe I shouldn’t say this, but we find a compromise to be able to say goodbye to that child over another two days, to leave them on a ventilator, to turn off all inotropic support, maybe to leave some minimal infusion, I think it’s not right, but we give them time to say goodbye… We know that there is no resuscitation for terminal hematological patients…*

*…We have these cases of cerebral injury where the EEG is practically a straight line, and the parents want resuscitation…*

However, physicians found it more difficult to withdraw treatment already started than to withhold treatment.

*…It’s a lot harder when we withdraw what has been started in the hope that the patient will be better… it’s always mentally harder for me because you know the battle you started is lost. When you withhold you don’t even start because you know that the patient doesn’t need anything. When you stop dialysis or inotropes in the course of intensive treatment, or take them off the ventilator because the patient is unpromising, it is much harder for me…*

*…for me personally, it is easier not to take action if we have agreed, come to some kind of consensus. Then I have an attitude towards it, and I know the patient in front of me, I know what I need or don’t need to provide for them. It is much harder… to cancel a treatment, whether it is antibiotic therapy or some form of non-invasive support that has already been provided, so withdrawing it is much harder for me…*

The need for palliative care is recognized, but it is often provided by the same healthcare staff. In other words, there are no distinct palliative care services in this setting. Healthcare professionals view this as an integral part of their everyday work, and their comprehensive care for the patient and their loved ones. The need for better regulation of end-of-life practices was also expressed:

*It would be much easier if the legislature had some sort of stance on it, and if there was something that made it easier for you, some sort of mechanism of action.*

Physicians who spent some time abroad made concrete comparisons between the Croatian situation and the situation in other countries:

*…In USA where I went for education… in the ICU there is a clear written DNR notice, or do not put on a ventilator… there are certain committees where the expert in this field, an ethicist, and a lawyer discuss this with you so that this is not only your decision…*

#### 3.2.4. “Spill-Over”

The high burden and stakes regarding the burden of making definite decisions was commonly expressed by physicians, particularly by those working in NICUs. Many different, individually specific strategies were used in order to tackle the enduring and cumulative consequences of such decisions.

*It’s hard for you, for days the agony lasts, for days you question, listen to yourself, juggle between different options and decisions. The moment I make a decision… I am calm… in some way relieved, sure that I have gone through all the options 1000 times and that I have made a good decision, no matter how difficult it is.*

Medical leaders commonly expressed the need to “stay strong” in order to comfort and support all the other individuals involved, which involves significant emotional effort (both on superficial and deeper levels) and dissonance. The importance of adequate formal and informal networks for emotional, spiritual and ethical support was emphasized, as well as the need for adequate time for reflection (commonly referred to as a “sacred pause”).

*I would like an ethical commission to take part in these decisions. This is just formalism in our country, because I have been to them two or three times and they just said that they had nothing to do with it. In my opinion, both a lawyer and a priest should be involved… We agree on everything here, but I think that these are big and difficult decisions, and that someone else from the outside should take part in them…*

The importance of meaningful activities and life outside the ICU was emphasized, but it was also stressed that their work has a high “spill-over” effect into their personal lives.

The needs of healthcare providers are perceived as being largely disregarded, and there is an omnipresent lack of the training and skills necessary to tackle all the possible negative consequences of their everyday work.

*I would like to have a meeting on this topic at the Croatian level once a year… of course we have meetings… but I would like to actually discuss individual cases, feelings, attitudes, with someone who fully understands our situation and has the same dilemmas as us. It’s hard for me to talk to an endocrinologist when he never has such a dilemma. Someone who is from the same field as us has similar dilemmas…*

#### 3.2.5. The Four Walls of the ICU

The existence of different microsystems of care delivery was strongly emphasized, as when critically ill children recover from their illness, significant organ dysfunction frequently persists, leading to chronic and often complex medical issues, as well as technology dependence (e.g., ventilator dependence and/or dialysis dependence). In other words, neonatal and pediatric critical care are no longer considered to be practiced in isolation; instead, critical neonatal and pediatric illness lies along a continuum. This is perceived as not adequately addressed by policymakers and healthcare institution management, so healthcare professionals often have the feeling that they need to “stretch themselves” in order to provide adequate care in the face of multiple organizational shortcomings.

*We also have children with chronic conditions who are at home, whose parents do everything our staff does here. However, it is easier for those whose children are placed with us than for those who do all this at home… they have care 24 h a day, parents come for an hour or two, no matter how emotionally difficult it is, and then they go home… Apart from one special institution, we do not have a single institution where parents who cannot take care of their children at home, for various reasons, can leave them there in permanent care.*

Additionally, and this was most prominent in PICUs, intensive care specialists are commonly perceived as “the ones dealing with death issues” by other medical specialists.

The issue of improving conditions in the ICUs to provide adequate space and time for parents and children to say goodbye was mentioned. The importance of support for the grieving parents was also emphasized.

*…I would like that when a child is at the end of life, they can have their own intimate space, that they can have their own nurse and that the parents can be with their dying child, which is not the case with us. Because we don’t have space, we have a child in relatively good condition lying next to a dying child in the same space… parents don’t have peace, they can’t even mourn their child…*

There were also some mixed experiences related to the provision of psychological support both for parents and physicians, from very good experiences, which stress the importance of having a psychologist and psychiatrist on call, and the need to have them more, to an interesting opinion expressed by the head of a ward:

*We have psychologists, but I’m even crazier all day when I know that the psychologist and psychiatrist are coming. I felt as the head of the department that it was a waste of time. This may be completely wrong because probably others need them. I was selfish and I didn’t want to listen to any of the problems of my nurses and maids on the ward… I do not want to listen to what her husband does at home and what kind of problems she has, and then they start to cry. I had to leave the room every time because I’m the boss here and I really don’t want to listen to it. When the psychologist and psychiatrist come, I leave…*

## 4. Discussion

To our knowledge, this is the first study of decision-making related to the end of life in pediatric and neonatal ICUs in tertiary-level healthcare institutions in the Republic of Croatia, from the perspective of physicians and nurses. The focus group participants gave detailed accounts of their experiences and the challenges that they faced in their everyday work. There were some similarities and differences among different types of ICUs (NICUs and PICUs) and focus group participants.

The thematic analysis found agreement between the perceptions and experiences of end-of-life issues among physicians and nurses within NICUs and PICUs. This was most prominent in the interactions with the families and communication with family members. The importance of the child’s best interest in a situation of vulnerability and medical prognostic uncertainty was stressed. The understating of the various values and goals of healthcare professionals and families, when it comes to treatment and end-of-life decision-making, was expressed as in previous studies [32,33,34,35,36]. Different approaches to coming to an understanding and mutual agreement were also deemed important, keeping in mind the special cultural circumstances in Croatia. In this context, the spiritual needs of family members and children were highlighted several times. This is not unusual since Croatia is a country with a significant number of religious people, according to the 2011 census (91.37% members of Christian denominations, of which 86.28% are Catholic, 1.44% Muslims and 0.01% Jewish).

This is an interesting observation if we compare our finding to the findings of the parallel focus group research that we undertook as a part of the VAL-DE-END project. In focus group discussions in adult ICUs in Croatia, we found significant differences in the perceptions and experiences of healthcare professional groups. Physicians working in adult ICUs were more focused on the lack of legal frameworks guiding their decision-making, and the different views of patients, family members and medical staff and within the medical team about end-of-life issues. Healthcare and organizational aspects and professional relationships with other members of the team and within the institution were also highlighted. The uniqueness, in a clinical sense, of each patient was stressed, as well as the role of the physicians in initiating the end-of-life discussions and decision-making. Nurses, on the other hand, were more focused on patient experience and vulnerability, their own personal experiences and involvement with patients, emotional burdens experienced in the process and the role ambiguity and interpersonal communication issues. The nurses were more inclined to stress the importance of personal values and beliefs when engaging in end-of-life decision-making. This difference in the perceptions and experience of physicians and nurses was not present in PICUs and NICUs. It seems that physician and nurses in PICUs and NICUs are all in the “same boat” when it comes to the issues and burdens that they experience [36].

The most prominent difference between the professional groups was the emphasis that nurses placed on the importance of adequate interprofessional relationships, while physicians placed more emphasis on intraprofessional relationships. Similar results may be found in the scientific literature regarding this topic, but also within the broader literature on professional relationships [5,6]. Despite the nurses’ emphasis on interprofessional relationships, it seems that their input in the decision-making process about end-of-life issues in Croatian NICUs and PICUs is highly valued, facilitated and appreciated by the physicians in the healthcare team. Physicians’ emphasis on intraprofessional relationships has several layers. The most dominant discussion was about consensus when it comes to decision-making among different physicians working in the ICU, and how this influences the care of the patients. The different conceptions that physicians have about what constitutes futile care were also stressed as commonly contributing to the inconsistencies of end-of-life procedures [15,37,38,39]. Physicians in PICUs had problems with other colleagues’ views on their units as places where children die quite often. Such misconceptions can give rise to various issues regarding end-of-life care, and ultimately contribute to the unnecessary initiation of potentially inappropriate treatment interventions [7,37,40].

High variability in the procedures used in end-of-life care was also found between different units and institutions. This indicates the need for developing and implementing clinical and professional guidelines [35,38,40,41]. Several units reported having specific local guidelines. However, decision-making was mainly based on certain unwritten rules. Previous studies conducted in other settings also found considerable variability regarding the management of end-of-life care and related issues in the pediatric critical care setting [38,40,41]. However, a need for additional ethical support services, specialized meetings and adequate decision-making guidelines was expressed by the focus group participants. The importance of having adequate facilities (e.g., secluded spaces for families whose child is dying) and of having up-to-date technical equipment was highlighted.

In our research, physicians did not consider withdrawing or withholding treatment interventions as morally equivalent procedures. Considering the previous literature, this is quite an interesting finding, as, in most of the literature, withdrawing and withholding are considered to be equivalent [16,42,43,44,45,46,47]. Participants placed a significant emphasis on the negative psychosocial consequences associated with everyday clinical work, facilitated by the complexities involved in end-of-life issues. The antecedents and factors influencing such notions as emotional fatigue, exhaustion, distress and burnout varied between professional groups, as well as between units. Such experiences seem to occur at higher levels and have more devastating effects on healthcare staff working in NICUs. These findings are in line with the growing recognition that the focus should shift to the physical, mental and spiritual well-being of healthcare providers, in order to improve the quality of care and the patients’ and parents’ experience [2,4,12,13,19,46,47,48]. However, although the majority of participants stressed the need for and importance of psychological support for both healthcare workers and family members, some of the participants were less enthusiastic about their implementation in the everyday work of an ICU.

This study has several limitations. Although the purposive sampling method has limitations, it is often considered adequate in studies using qualitative methods, especially if the target population of participants is not large and the topic itself is rich. We used professionally homogenous focus groups in order to create a safer environment for the study participants, and to facilitate discussion on this value-laden topic. The generalizability of the findings is limited since one institution (Osijek Clinical Hospital Center) at the tertiary level of healthcare in the Republic of Croatia was not included since ethical committee approval was not obtained.

## 5. Conclusions

The perceptions and experiences of end-of-life issues among nurses and physicians working in NICUs and PICUs in the Republic of Croatia share multiple common characteristics. Firstly, the interrelatedness of the high emotional and cognitive demands, and the high burden associated with end-of-life issues in this setting, have a significant influence on the personal and professional lives of healthcare professionals. Therefore, there is a need for developing psychological support services and ethical counselling services for physicians and nurses in order to address these burdens and prevent burnout. Secondly, not only physicians and nurses but families and patients also experience different emotional and decision-making burdens. The medical staff that work with the families and patients in PICUs and NICUs in Croatia should therefore possess adequate knowledge about different aspects of end-of-life care in order to help families and patients to cope with the consequences of certain decisions. Additional support (psychological, spiritual and social) should also be available for families and patients to help them to navigate through end-of-life situations. Technical and organizational shortcomings were also described by the participants of this study. Therefore, the improvement of existing facilities and equipment is an important prerequisite for better provision of care. Finally, the findings about the high variability of end-of-life procedures applied, and the various difficulties experienced during the shared decision-making process, underline the need for developing clinical and professional guidelines and the need to influence policymakers. The VAL-DE-END project, of which this focus group research is a part, has the drafting of possible end-of-life guidelines for the ICU setting in Croatia as one of its outputs. At the end of the project, when all the research has been analyzed, the interdisciplinary project team will try to draw up draft guidelines based on all the research findings. Moreover, within the project, we have already conducted a systematic review of the ethical content of expert recommendations for end-of-life decision-making in adult, pediatric and neonatal ICUs. This analysis will help us with our efforts.

## Figures and Tables

**Table 1:** Focus group participants.

Research Site	Physicians	Nurses	Physicians		Nurses		Physicians		Nurses		Physicians		Nurses	
			Male	Female	Male	Female	<5 Years of Experience	5< Years of Experience	<5 Years of Experience	5< Years of Experience	NICU *	PICU *	NICU *	PICU *
Zagreb	8	9	3	5	-	9	1	7	4	5	5	3	6	3
Rijeka	6 (4 + 2)	7 (4 + 3)	1	5	-	7	3	3	3	4	2	4	3	4
Split	6	5	1	5	1	4	3	3	3	2	3	3	3	2
TOTAL	20	21	5	15	1	20	7	13	10	11	10	10	12	9

* neonatal intensive care unit (NICU); pediatric intensive care unit (PICU).

**Table 2:** Focus group discussion guide.

Discussion Subsets	Discussion Structure
A. General introduction	General introduction into focus groups discussion and explanation
B. Opening	Let’s start by telling us your name, years of service and how many of your colleagues do you share your shift on a normal working day?
C. Introduction	You’ve probably heard the term end-of-life decisions often, but what exactly does that term mean to you? What do you mean by cessation of active treatment? - possibilities (renunciation/non-initiation, interruption/cessation => cessation of active treatment; palliative care; conscious, active, intentional action with the purpose of killing/cessation of life => active shortening of life) - procedures (resuscitation, artificial ventilation; extubation; antibiotics; hydration => ordinary/usual—extraordinary/unusual)
D. Transition	How often do you encounter this in your daily work? Can you give examples of situations you have encountered?
E. Main discussion	**1. Discussion and decisions** What most often triggers a discussion about cessation of active treatment (renunciation/cessation) or end-of-life decisions? Who most often initiates a discussion? Who leads it, encourages it? Who is participating in the discussion? Who usually decides to stop active treatment? What are individuals guided by when deciding to discontinue active treatment? - patients, family members, legal representatives; doctors; nurses; someone else [e.g., ethics committee, court] How much is your opinion valued? How is the opinion of the patient, his relatives or legal representatives evaluated? other physicians nurses What causes disagreement in end-of-life decisions? What do you do when you do not agree with the decision to stop active treatment? What do you do when you think your current treatment is futile? What do you do when you think that the wish of the patient or his relatives is unfounded? How often does it happen that it is necessary to revise an already made decision? **2. Implementation of the decision** What are the most common problems you encounter with cessation of active treatment? Can you give examples that you have encountered? Have you ever found yourself in a situation where you did not know what to do? Please describe the situation. How did you feel? Did you have support? Do you think something should be improved in intensive care units regarding cessation of active treatment and end-of-life decisions? What would it be? **3. General questions** Do you think that giving up/not starting, stopping/stopping active treatment is (ethically) identical procedures? Do you think that the procedures of active shortening of life in the hopelessly ill are ethically justified? What are all the pros and cons of actively shortening life in hopelessly ill people?
F. Conclusion	Is there anything else important that we haven’t talked about so far? Of all the things we talked about, what do you consider the most important?
G. Giving thanks	Thanks again for participating. I hope it was not overly demanding and that you enjoyed it. I remind you once again that the confidentiality of this conversation is absolute and I ask you not to share everything you have heard here today from your colleagues with others outside this group.

**Table 3:** Overview of main themes, subthemes and distant codes.

Main Theme	Subtheme	Codes
Critical illness	Child	uncertainty, best interest of the patient, recognition of suffering, awareness of futility, vulnerability
	Family	understanding, comprehension, presence, involvement, communication, expectations, handing over care, specific needs, competing interests of parents and patients
	Myself	being engaged and distanced/emphatic, detached, distress, emotional effort, emotional dissonance, compassion fatigue, burnout, exhaustion, (disenfranchised) grief, secondary victimization, closure
	Other professionals	value and importance of input, communication strategies, professional hierarchies, handling and sharing responsibility, microsystems of care, shared goals, understanding and perceptions
End-of-life	Breaking point	curative-intensive-palliative-end-of-life transition of care, initiation of end-of-life discussion
	Decision-making	interrelatedness of cognitive and emotional, the role of underlying values, understanding complex issues, communicating uncertainties, withholding information about futile treatment possibilities, definitions and the process of determination of death
	End-of-life procedures	withdrawing, withholding, basal therapy, symbolic care, palliative care, legal background
	“Spill-over”	burden of definite decisions, personal strategies, resilience, importance of outer life, lack of recognition, importance of leadership, skills and training, emotional and instrumental support
	Four walls of the ICU	intensive care outreach, continuity of care, complex needs of survivors, misconceptions about ICUs, technical/organizational shortcomings

## Data Availability

The data presented in this study are available on request from the corresponding author. The data are not publicly available due to privacy reasons.
